# Noninvasive Monitoring of Placenta-Specific Transgene Expression by Bioluminescence Imaging

**DOI:** 10.1371/journal.pone.0016348

**Published:** 2011-01-21

**Authors:** Xiujun Fan, Peigen Ren, Sabita Dhal, Gill Bejerano, Stuart B. Goodman, Maurice L. Druzin, Sanjiv S. Gambhir, Nihar R. Nayak

**Affiliations:** 1 Department of Obstetrics and Gynecology, Stanford University School of Medicine, Stanford, California, United States of America; 2 Department of Orthopaedic Surgery, Stanford University School of Medicine, Stanford, California, United States of America; 3 Department of Computer Science, Stanford University, Stanford, California, United States of America; 4 Molecular Imaging Program at Stanford, Departments of Radiology and Bioengineering, Bio-X Program, Stanford University School of Medicine, Stanford, California, United States of America; Institute of Zoology, Chinese Academy of Sciences, China

## Abstract

**Background:**

Placental dysfunction underlies numerous complications of pregnancy. A major obstacle to understanding the roles of potential mediators of placental pathology has been the absence of suitable methods for tissue-specific gene manipulation and sensitive assays for studying gene functions in the placentas of intact animals. We describe a sensitive and noninvasive method of repetitively tracking placenta-specific gene expression throughout pregnancy using lentivirus-mediated transduction of optical reporter genes in mouse blastocysts.

**Methodology/Principal Findings:**

Zona-free blastocysts were incubated with lentivirus expressing firefly luciferase (Fluc) and Tomato fluorescent fusion protein for trophectoderm-specific infection and transplanted into day 3 pseudopregnant recipients (GD3). Animals were examined for Fluc expression by live bioluminescence imaging (BLI) at different points during pregnancy, and the placentas were examined for tomato expression in different cell types on GD18. In another set of experiments, blastocysts with maximum photon fluxes in the range of 2.0E+4 to 6.0E+4 p/s/cm^2^/sr were transferred. Fluc expression was detectable in all surrogate dams by day 5 of pregnancy by live imaging, and the signal increased dramatically thereafter each day until GD12, reaching a peak at GD16 and maintaining that level through GD18. All of the placentas, but none of the fetuses, analyzed on GD18 by BLI showed different degrees of Fluc expression. However, only placentas of dams transferred with selected blastocysts showed uniform photon distribution with no significant variability of photon intensity among placentas of the same litter. Tomato expression in the placentas was limited to only trophoblast cell lineages.

**Conclusions/Significance:**

These results, for the first time, demonstrate the feasibility of selecting lentivirally-transduced blastocysts for uniform gene expression in all placentas of the same litter and early detection and quantitative analysis of gene expression throughout pregnancy by live BLI. This method may be useful for a wide range of applications involving trophoblast-specific gene manipulations in utero.

## Introduction

Placental dysfunction underlies numerous complications of pregnancy affecting both maternal and fetal health [1], [2]. Over the past two decades, transgenic and knockout studies in the mouse have substantially advanced our knowledge of the genetic control of placental development [2]. However, although the recent development of trophoblast lineage-specific lentivirus infection system appears to be highly promising for placenta-specific gene manipulation [3]–[6], there are several major shortcomings. Considerable variability in gene expression has been reported among different placentas of the same litter, which would make the interpretation of results difficult [4], [6]. Furthermore, the extent of gene expression is determined by histological examination of the placenta at term while many genes are expressed only at specific stages of placental development, not consistently throughout pregnancy, underscoring the critical need for an efficient, noninvasive method of monitoring gene expression at different stages of placental development.

Recent advances in molecular imaging techniques provide a unique capability for noninvasive and serial monitoring of gene expression in the same living animal [7]. Of the various imaging modalities in use, BLI with light-emitting enzymes (luciferases) provides a relatively simple, sensitive, and low-cost alternative to study reporter gene expression in small animal models [7], [8]. Luciferin, the substrate for luciferases, rapidly diffuses through most tissues and is relatively stable in vivo providing long-lived luminescent signals [7]. In addition, the nonimmunogenic characteristics of luciferin make this method ideally suited for repeated *in vivo* imaging [7], [8]. Numerous studies show imaging of bioluminescent Fluc reporter gene expression by adenovirus- and lentivirus-mediated gene transfer into various organs [9]–[12]. BLI of Fluc has been successfully used to monitor changes in gene expression associated with discrete biological processes, including the responses to chemical stress, tumor hypoxia and heat shock [13]. Also, with the rapid expansion of this technology in recent years, various luciferases have been programmed to detect specific protein functions, phosphorylation events [14], and bioactive small molecules [13]. However, a versatile, rapid, and sensitive assay to study gene functions in the placenta in intact animals has not been described.

In the present study, we developed a sensitive method for repetitively tracking transgene expression in the mouse placenta throughout pregnancy. We showed that expression of Fluc in trophoblasts and repeated exposure to its substrate (luciferin), either at the blastocyst stage or during pregnancy, had no adverse effect on blastocyst viability or continuation of pregnancy. We then confirmed the feasibility of uniform gene expression in all placentas of the same litter by selecting optimally lentiviral transduced blastocysts - which is essential for quantitative and noninvasive monitoring of gene expression at different stages of pregnancy.

## Results and Discussion

**Figure 1:**
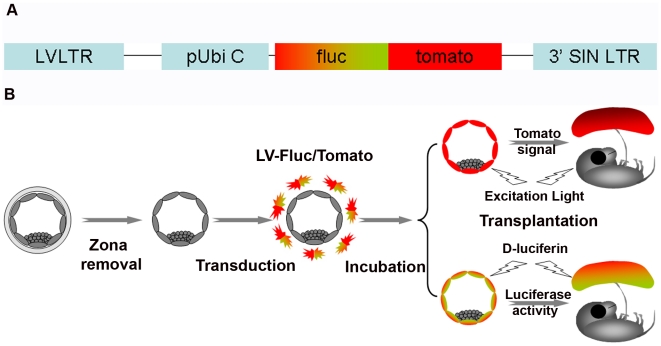
Diagrammatic representation of the lentivirus vector construct and trophoblast-specific lentiviral gene delivery. A, the lentiviral double-fusion reporter gene construct (LV-Fluc/Tomato). Fluc/Tomato was cloned downstream of the ubiquitin C (Ubi C) promoter with a 14-amino acid (LENSHASAGYQAST) linker. B, zona-free blastocysts were transduced with LV-Fluc/Tomato and transduction efficiency of each blastocyst was evaluated by BLI and Tomato fluorescence. Optimally transduced blastocysts were then transferred into pseudopregnant recipients. Fluc expression in the placenta was assessed by BLI at various stages of gestation following intraperitoneal injection of D-luciferin, and Tomato expression in different cell types was assessed after collection of placentas on GD18. Note that these strategies permit quantitative assessment of placenta-specific transgene expression in the same animal at different stages of pregnancy.

For both live imaging and examination of cell-specific expression of reporter genes, we used a lentiviral vector expressing Fluc-Tomato fluorescent fusion protein driven by the constitutive ubiquitin C promoter (LV-Fluc/Tomato) (Fig. 1). First, we determined the optimum LV-Fluc/Tomato titer, virus incubation time, and luciferin dose for live imaging of LV-Fluc/Tomato-transduced zona-free blastocysts. The transduced blastocysts were transplanted into day 3 pseudopregnant recipients (GD3). Similar to earlier reports in mice and rats, higher viral titers (greater than 1.25×10^10^ particles/ml) and prolonged incubation times (18 hours or longer) resulted in a lower rate of implantation (35.29% and 33.33%, respectively)[4], [6]. However, brief exposure to increased doses of luciferin before blastocyst transfer had no marked effect on implantation, and 50 µg luciferin/ml of KSOM was used in this study, based on a luciferin dose-response curve (maximum photon flux/blastocyst). Similarly, short exposure of blastocysts for examination of tomato fluorescence did not affect the implantation rate (60.71%).

**Figure 2:**
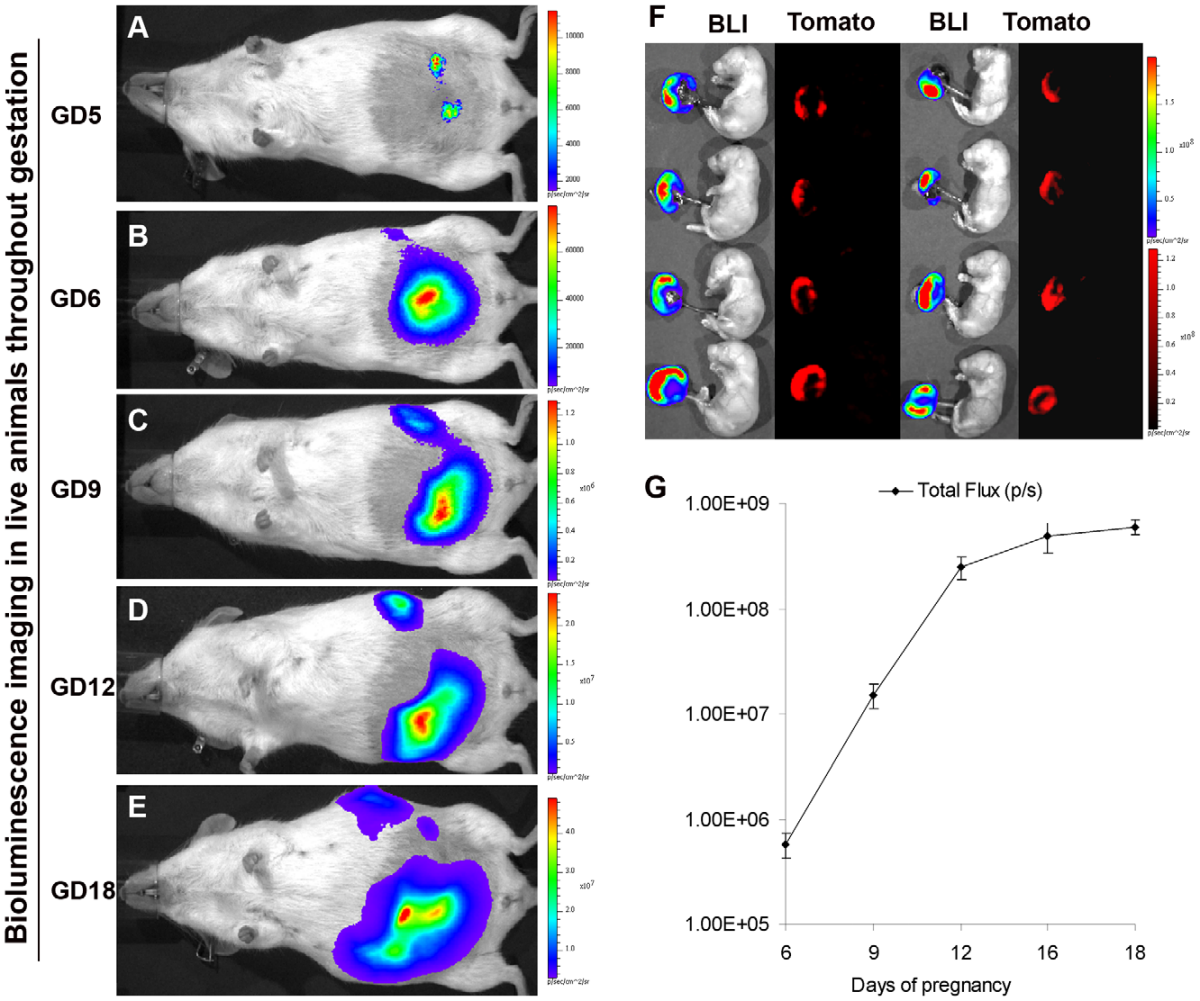
Trophoblast-specific Fluc expression assessed by live BLI at different stages of pregnancy. Blastocysts (selected) optimally transduced with LV-Fluc/Tomato were transferred into GD3 pseudopregnant recipients and Fluc expression in the placenta was evaluated by BLI at different stages of pregnancy in the same animal. A–E, grayscale body surface images and pseudocolor luminescence images (blue - least intense, red - most intense) were superimposed; photons emitted from implanting blastocysts could be detected as early as GD5 (A). F, placenta-specific Fluc (BLI) and Tomato (fluorescence) expression on GD18. Note that fetuses of the corresponding placentas are both Fluc and Tomato negative, indicating viral transduction of trophoblast-specific lineage. G, levels of total photon flux over the abdominal area at different stages of pregnancy; there was an exponential increase in signal intensity from GD6 through GD12.

We next evaluated the feasibility of *in vivo* BLI of Fluc expression by transplanted blastocysts at various stages of pregnancy using the optimized virus titer (1.25×10^10^ particles/ml) and incubation time (4 h). The recipients were imaged for Fluc activity immediately after blastocyst transfer (about 2PM) on GD3, then every 6 hours starting at 6 AM of GD4 till 6PM of GD6, and subsequently at 2PM on GDs 9, 12, 16 and 18. The placentas and fetuses were collected on GD18 for both live imaging and examination of tomato expression in different cell types. Fluc expression was detectable on the abdominal surface in the surrogate dams at 6PM on day 5 of pregnancy (within two days after blastocyst transfer) by live imaging (Fig. 2A), indicating a high sensitivity of detection of bioluminescence signals from implanting blastocysts in live animals during early pregnancy. Since signals from blastocysts transferred with luciferin into the uterine lumen were also not detectable by live BLI (Figure S1), and as implantation usually occurs on GD5, it is likely that the number of photons emitted from preimplantation blastocysts and transmitted through the uterine and abdominal walls were below the detection limit for live BLI. The signal is probably detectable only after rapid expansion of trophoblast cells at the beginning of implantation. Although hypoxia is known to interfere with luciferase oxidation and BLI, our results indicate that the signals from implanting blastocysts during early pregnancy can be detected by BLI despite the intensely hypoxic environment.

**Figure 3:**
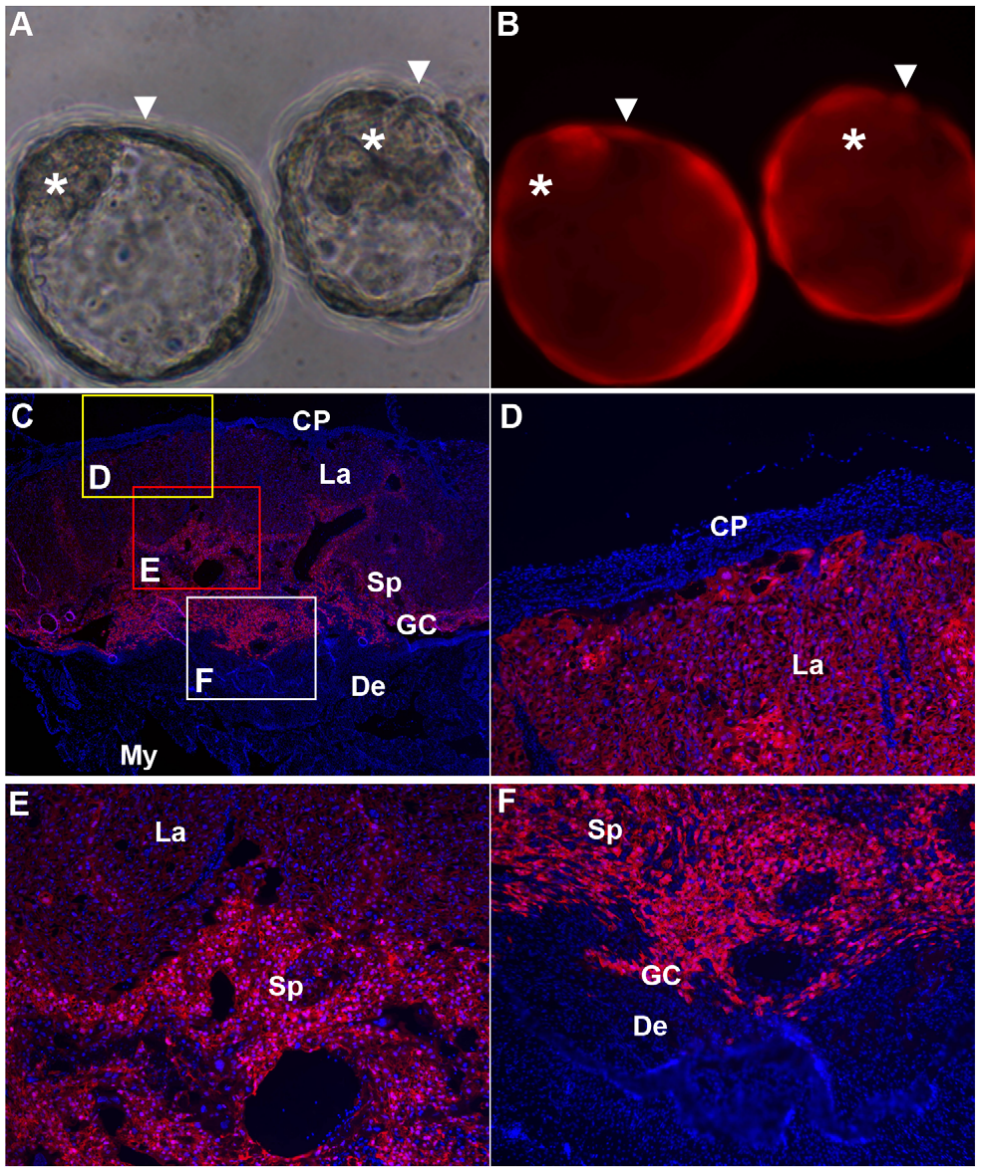
Trophoblast-specific Tomato expression by lentivirus-mediated transgene delivery into blastocysts. Tomato expression was examined in zona-free blastocysts infected with LV-Fluc/Tomato (A–B) and in GD18 placentas (C–F) after blastocyst transfer into pseudopregnant mice. A–B, phase-contrast (A) and fluorescence (B) images showing Tomato expression in the trophectoderm of blastocysts (A, B, ▾) and not in the inner cell mass (A, B, *). C–F, Tomato expression in trophoblast lineages of placentas; D–F, magnified areas in C; giant cells (GC), spongiotrophoblast (Sp) and labyrinth (La) layers, decidua (De), myometrium (My), and chorionic plate (CP).

The photon flux measured on the abdominal surface increased exponentially after GD5 until day 12, reaching a peak level at day 16 and maintaining that level until day 18 (Fig. 2A–E, G). All of the placentas, but none of the fetuses, analyzed on day 18 by BLI showed Fluc expression (Fig. 2F). Although the molecular mechanism is not clearly understood, it has been shown that the trophectoderm layer can serve as a robust barrier to lentivirus particles and protect the inner cell mass (ICM) from virus infection [15], and E-cadherins and tight junctions are suggested to contribute to this barrier function [16], [17]. Tomato expression in placentas was observed in all trophoblast cell lineages (Fig. 3); however, it was not uniform across all trophoblast cell lineages, with more intense expression occurring in spongiotrophoblasts and giant cells (Fig. 3). Consistent with previous reports [4], [6], the trophoblast lineage-specific differences in gene expression suggests distinct transcriptional environments in different lineages. Furthermore, we did not observe any significant differences in the rates of implantation or the numbers of live fetuses and resorption sites between pregnancies with zona-free LV-Fluc/Tomato-transduced blastocysts and nontransduced blastocysts (Table 1), indicating that the lentiviral vector and multiple BLI have no significant effect on the pregnancy outcome.

**Table 1:** Effect of lentivirus transduction (LV-Fluc/Tomato) into blastocysts and live bioluminescence imaging on pregnancy outcome.

	Viral titer (particles/ml)	Blastocysts transferred (Number)	Implantation (%)	Live fetus (%)	Resorption sites (%)
Control	0	60	63	47	17
LV-Fluc/Tomato	1.25×10^10^	78	63	45	18

**Figure 4:**
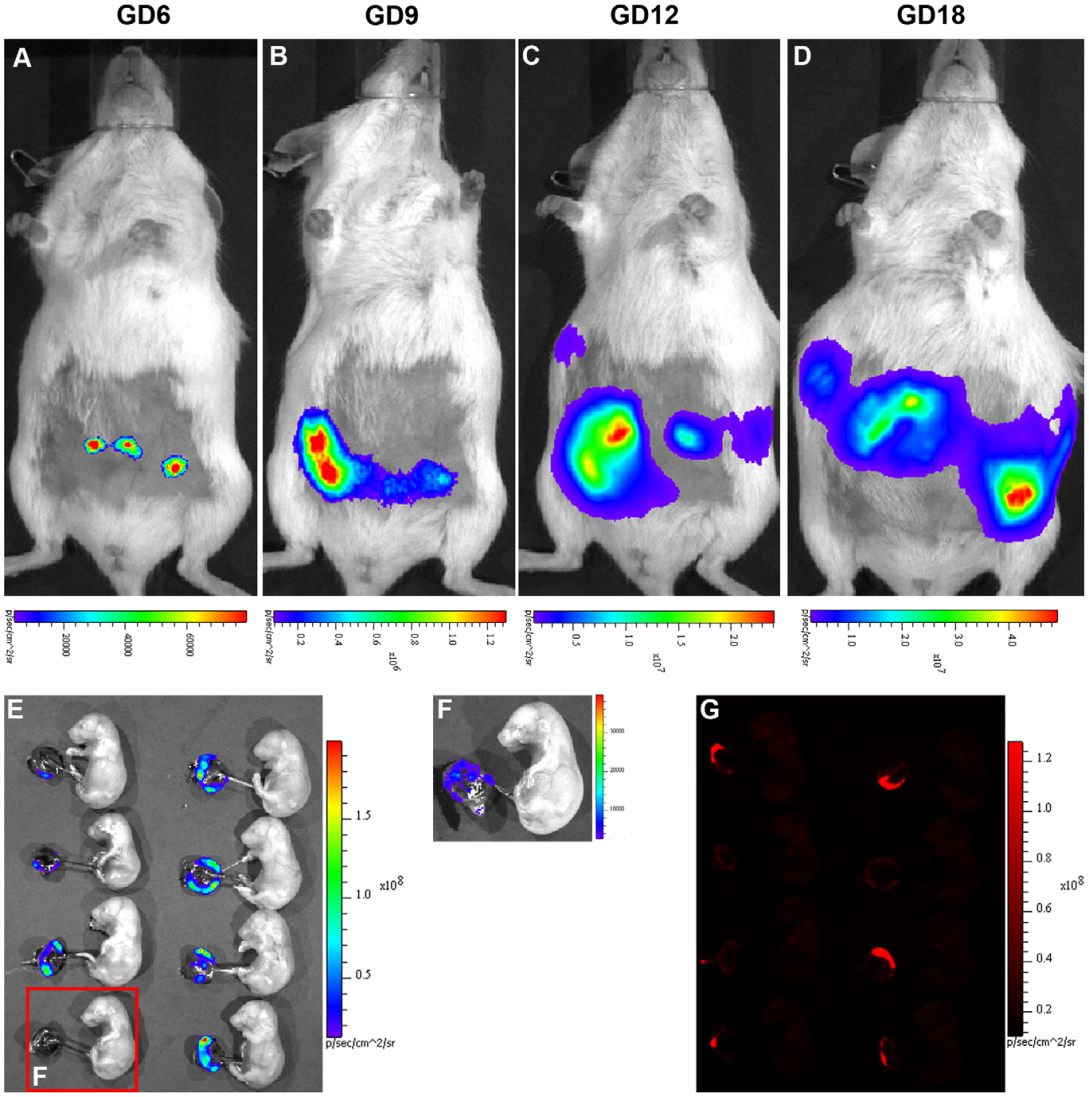
Wide variability in Fluc expression among placentas of the same litter despite identical conditions of viral transduction of blastocysts. A–D, BLI of Fluc expression in placentas at different stages of pregnancy; E, dramatic variations in Fluc expression among different placentas from the same litter collected on GD18; F, detection of very weak Fluc signal in a placenta (marked in E) after lowering the threshold of BLI. G, Tomato florescence images of the placentas shown in E.

However, similar to published reports[4]–[6], despite the same conditions of viral transduction, there was considerable variability in both Fluc and Tomato expression between placentas of the same litter (Fig. 4 E–G). In a separate experiment, we observed marked increase in Fluc intensity in late-stage compared to early-stage blastocysts transduced with the same titer of LV-Fluc/Tomato (data not shown). Thus, we believe that, although morphologically indistinguishable, minor differences in developmental stages of blastocysts may contribute significantly to the variability in transgene expression in placentas of the same litter.

**Figure 5:**
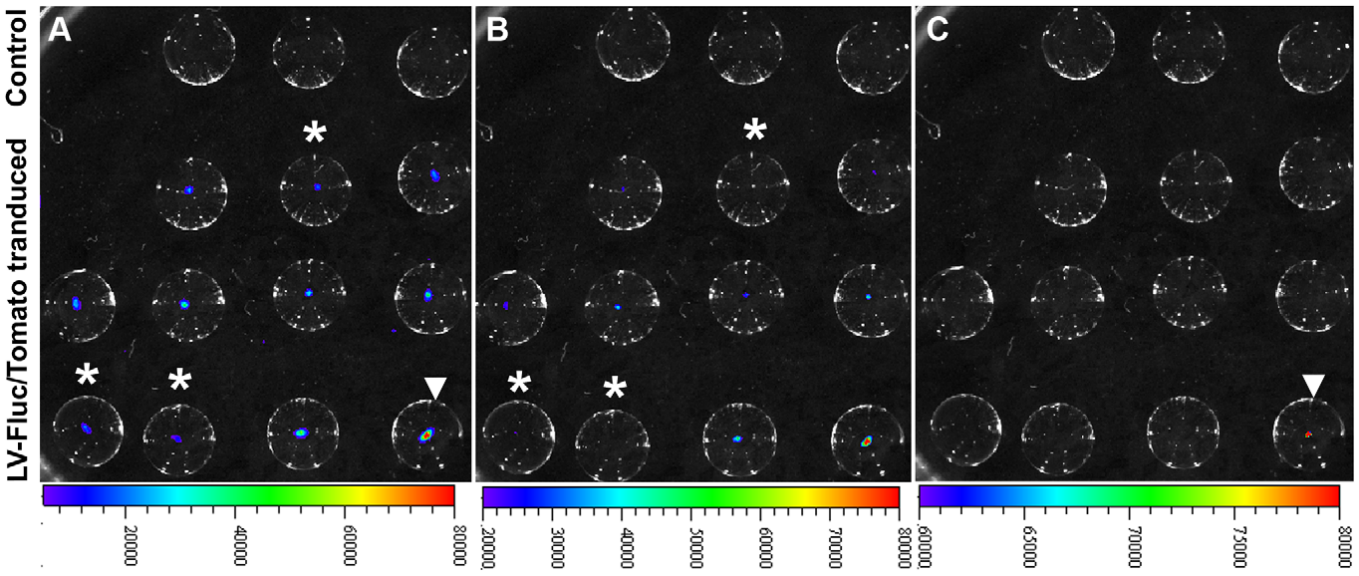
Selection of blastocysts for optimal lentivirus transfection efficiency. Each LV-Fluc/Tomato transduced blastocyst was incubated in KSOM containing D-luciferin (50 ug/ml) and assessed for Fluc expression (luciferase activity) by BLI. A, wide variations in Fluc signals from different blastocysts despite identical virus transfection conditions; B–C, blastocysts with BLI values between 2.0E+4 and 6.0E+4 p/s/cm^2^/sr were selected for transfer. Increase of BLI threshold to 2.0E+4 p/s/cm^2^/sr identifies blastocysts with low signals (B, *), and further increase in threshold to 6.0E+4 p/s/cm^2^/sr identifies blasocysts with very high intensity signals (C, ▾).

We next examined the feasibility of preselecting blastocysts for uniform gene expression in all placentas of the same litter. LV-Fluc/Tomato-transduced blastocysts were examined for levels of Fluc expression by BLI, and based on the results of a pilot study (Figure S2), blastocysts within the range of 2.0E+4 to 6.0E+4 photons per second per centimeter square per steradian (p/s/cm^2^/sr) (Fig. 5) were transferred into recipients. Total Fluc activity in each animal was measured by live BLI on GDs 6, 9, 12, 16 and 18, and the placentas and fetuses were collected on GD18. Except for one placenta from a highly growth-restricted fetus (out of 5 litters), total photon flux/placenta varied between 1.85E+8 p/s and 4.55E+8 p/s in all litters, and there were no significant differences in total photon flux between the placentas from the same litter or placentas from different litters (Fig. 2). Moreover, except at GD6 (*r* = 0.58), total photon intensity in each pregnancy was significantly correlated with the number of placentas at GDs 9 (*r* = 0.83), 12 (*r* = 0.93), 16 (*r* = 0.94), and 18 (*r* = 0.90). Therefore, spatiotemporal information on placenta-specific gene expression could be directly visualized noninvasively and quantified at different stages of pregnancy.

Our results demonstrate the first proof-of-principle study for the feasibility of quantitative analysis of gene expression in the placenta throughout pregnancy by live imaging. In addition to monitoring gene expression, using advanced BLI techniques and engineered bioluminescent probes, this method may be useful for a wide range of applications involving trophoblast-specific gene manipulations in utero, including the study of discrete biological functions and the detection of protein functions and other post-translational modification events in the placentas of living animals.

## Materials and Methods

### Animals

All animal experiments were conducted in the research animal facility at Stanford University with approved protocols from the Administrative Panel on Laboratory Animal Care (Protocol ID#12340). 8-10 week old CD-1 (Charles River, Wilmington, MA) female mice were mated with fertile or vasectomized males the same strain (10–16 weeks) to induce pregnancy or pseudopregnancy, respectively [18]. The day of detection of the vaginal plug was considered as day 1 of pregnancy/pseudopregnancy (GD1). Blastocysts from GD4 mice were collected for lentivirus transduction and transferred back into GD3 pseudopregnant mice as previously described [19], [20]. The surrogate dams were examined for Fluc expression by live BLI at different stages of pregnancy, and the placentas and fetuses were collected on GD18 for BLI, fluorescence imaging, and other histopathological analyses [18].

### Lentiviral Vector Production

The HIV-1-based self-inactivating lentiviral vector, LV-Fluc/Tomato, was produced following the same protocols as described previously [21]. Briefly, firefly luciferase [22] and tdTomato (from Dr. Roger Tsien, University of California San Diego) [23] cDNA fragments were cloned into pcDNA3.1 (Invitrogen, CA) for construction of the fluc2-tomato plasmid using Nhe I and Xho I, and EcoRI and BamHI restriction sites, respectively. The pLV-fluc-tomato (double-fusion gene) plasmid was generated using the NheI and BamHI fragment from the pcDNA 3.1 fluc2-tomato plasmid by blunt-end ligation into the multiple cloning site of the lentiviral transfer vector, FUW, driven by the human ubiquitin-C promoter [24]. Virus particles were generated by co-transfecting pLV-Fluc-Tomato plasmid, delta 8.9 packaging plasmid, and pVSV-G plasmids coding for VSVG envelope protein into 293T cells using the standard calcium phosphate method with chloroquine (final concentration 0.025 mM) [25]. After 48 hours of transfection, virus-containing supernatant was harvested, centrifuged at a low speed (2000 rpm for 10 min), and filter purified with a Millipore Stericup filter unit (Millipore, Billerica, MA). Virus particles were then concentrated using the PEG-it virus precipitation solution following the manufacturer's instructions (SBI, CA), resuspended in PBS, aliquoted and stored at -80°C. Virus titer (particles/ml) was determined using the QuickTiter Lentivirus quantitation kit (Cell Biolabs Inc, San Diego, CA).

### Embryo collection and viral transduction

Blastocysts were collected and transduced with LV-Fluc-Tomato following the same procedures reported before [5], [6]. Briefly, blastocysts were flushed with EmbryoMax M2 Medium (Millipore) on GD4 (8:30–10:00AM). After washing in microdrops containing KSOM Embryo Culture media (Millipore), the blastocysts were treated with acid Tyrode's solution (Sigma, Saint Louis, CA) for removal of zona pellucidae. For determination of optimal virus concentration and incubation time for blastocyst transduction, individual zona-free blastocysts were incubated in 5 ul KSOM drops containing different concentrations of LV-Fluc/Tomato (5.0×10^8^, 2.5×10^9^, 1.25×10^10^ and 6.25×10^10^ particles/ml) under light mineral oil (Irvine Scientific, Santa Ana, CA) for different duration of time (4, 6 and 18 hours). Transduced blastocysts were washed with M2 medium to remove extra viruses and transferred into GD3 pseudopregnant mice with or without evaluation of Tomato/Fluc expression.

### Bioluminescence imaging (BLI)

#### Selection of virus-transduced blastocysts by BLI

Individual blastocysts in culture plates were incubated in KSOM drops containing D-luciferin (50 µg/ml, Caliper, Alameda, CA) covered by light mineral oil. The concentration of D-luciferin was determined in a pilot experiment from peak photon fluxes by single blastocysts with different concentrations of D-luciferin. The plate containing the blastocysts was placed in a light-tight chamber of the Xenogen In Vivo Imaging System (IVIS 200, Caliper, Mountain View, CA), and photons emitted from each blastocyst (photons per second per centimeter square per steradian, p/s/cm^2^/sr) were measured. Based on the results of initial experiments, blastocysts with maximum photon fluxes in the range of 2.0E+4-6.0E+4 p/s/cm^2^/sr were selected for subsequent experiments.

### Live imaging of animals after blastocyst transfer

In vivo BLI in mice was performed using the Xenogen In Vivo Imaging System (IVIS 200) following the same protocols described previously [21]. The animals were maintained under isoflurane (1.5-2.5%) anesthesia throughout imaging. D-Luciferin (150 mg/kg body weight) was injected intraperitoneally (IP) into each animal five minutes prior to imaging. Fully anesthetized animals at different stages of pregnancy were placed in the imaging chamber with their shaved ventral surface facing the camera and snout positioned inside the nose cones attached to the anesthesia tubing. A grayscale body surface reference image was collected, and the photons transmitted through the tissues were acquired by the IVIS 200 for a set period of time (300, 60, 20, 10, and 1 sec integration times). Grayscale and pseudocolor luminescence images (blue least intense and red most intense) were then superimposed using the image-processing software Living Image 3.0 (Caliper Life Sciences). For data analysis, regions of interest (ROI) were defined over the uterine area, and total photon fluxes were quantified using Living Image 3.0 software. On GD 18, the animals were sacrificed immediately after live imaging, and the fetuses and placentas were removed and imaged under the IVIS 200 without additional D-luciferin administration.

### Fluorescence imaging

For examination of cell-specific Tomato expression, blastocysts in the culture media were directly examined under a phase contrast and fluorescence microscope (Zeiss Axioskop 2, Carl Zeiss, Oberkochen, Germany), and the images were captured and superimposed using a Zeiss AxioCam camera and Zeiss AxioVision 4.5 software (Carl Zeiss). Following BLI on GD18, Tomato expression in fetuses and placentas was examined using the IVIS 200 with appropriate fluorescence filters (DsRed, excitation 500–550 nm and emission 575–650 nm). The grayscale and fluorescence images were superimposed using Living Image 3.0 software. Tomato expression was then evaluated in different cell types of the placenta on GD 18. 10 µm frozen sections of placenta were fixed with 4% paraformaldehyde in PBS, washed in PBS, mounted in Vectashield mounting medium (Vector Laboratories, Burlingame, CA), and examined under the Zeiss Axioskop 2 florescence microscope.

### Statistical analysis

At least five samples in each experimental group were used for statistical analysis, and all data were expressed as means ± SE [18]. The significance of differences between means was analyzed by one-way ANOVA and t-tests using SPSS software (SPSS Inc, Chicago, IL, USA) [26], [27]. P values below 0.05 were considered significant. Correlations between different attributes (*r*) were calculated using SPSS software (SPSS Inc).

## Supporting Information

Figure S1 **Detection of Fluc expression by live BLI following transfer of LV-Fluc/Tomato-transduced blastocysts with D-luciferin.** Blastocysts in M2 medium containing D-luciferin (50 µg/ml) were transferred into GD3 pseudopregnant recipients, and Fluc expression was evaluated after IP injection of D-Luciferin (150 mg/kg body weight) into each animal by live BLI, immediately after blastocyst transfer (GD3 at 2PM, A) and again on GD4 (2PM, B) and GD5 (6PM, C). A–C, superimposed grayscale body surface images and pseudocolor luminescence images. Photons emitted from implanting blastocysts could be detected only on GD5 (C) - there was no detectable signal on GD3 (A) and GD4 (B).
(TIF)

Figure S2 **Variability in Fluc expression among placentas of the same litter after transfer of blastocysts with different BLI values.** LV-Fluc/Tomato-transduced blastocysts with BLI values in different ranges (1.0E+4 to 4.0E+4, above 3.0E+4, and 2.0E+4 to 6.0E+4 p/s/cm^2^/sr) were transferred to different groups of GD3 pseudopregnant recipients. A and B, placentas from recipients transferred with blastocysts of BLI values of 1.0E+4 to 4.0E+4 p/s/cm^2^/sr (A) and above 3.0E+4 p/s/cm^2^/sr (B). Placentas of transferred blastocysts having BLI values of 2.0E+4 to 6.0E+4 p/s/cm^2^/sr are presented in Figure 2F. Note that there is wide variability in Fluc expression among placentas in A (1.0E+4 to 4.0E+4 p/s/cm^2^/sr) and B (above 3.0E+4 p/s/cm^2^/sr), but not in Figure 2F (2.0E+4 to 6.0E+4 p/s/cm^2^/sr).
(TIF)
