# Supplementary figures and images for: Noninvasive Monitoring of Placenta-Specific Transgene Expression by Bioluminescence Imaging
Source: PLoS One. 2011 Jan 21;6(1):e16348. doi: 10.1371/journal.pone.0016348 (PMC3025029; doi:10.1371/journal.pone.0016348)

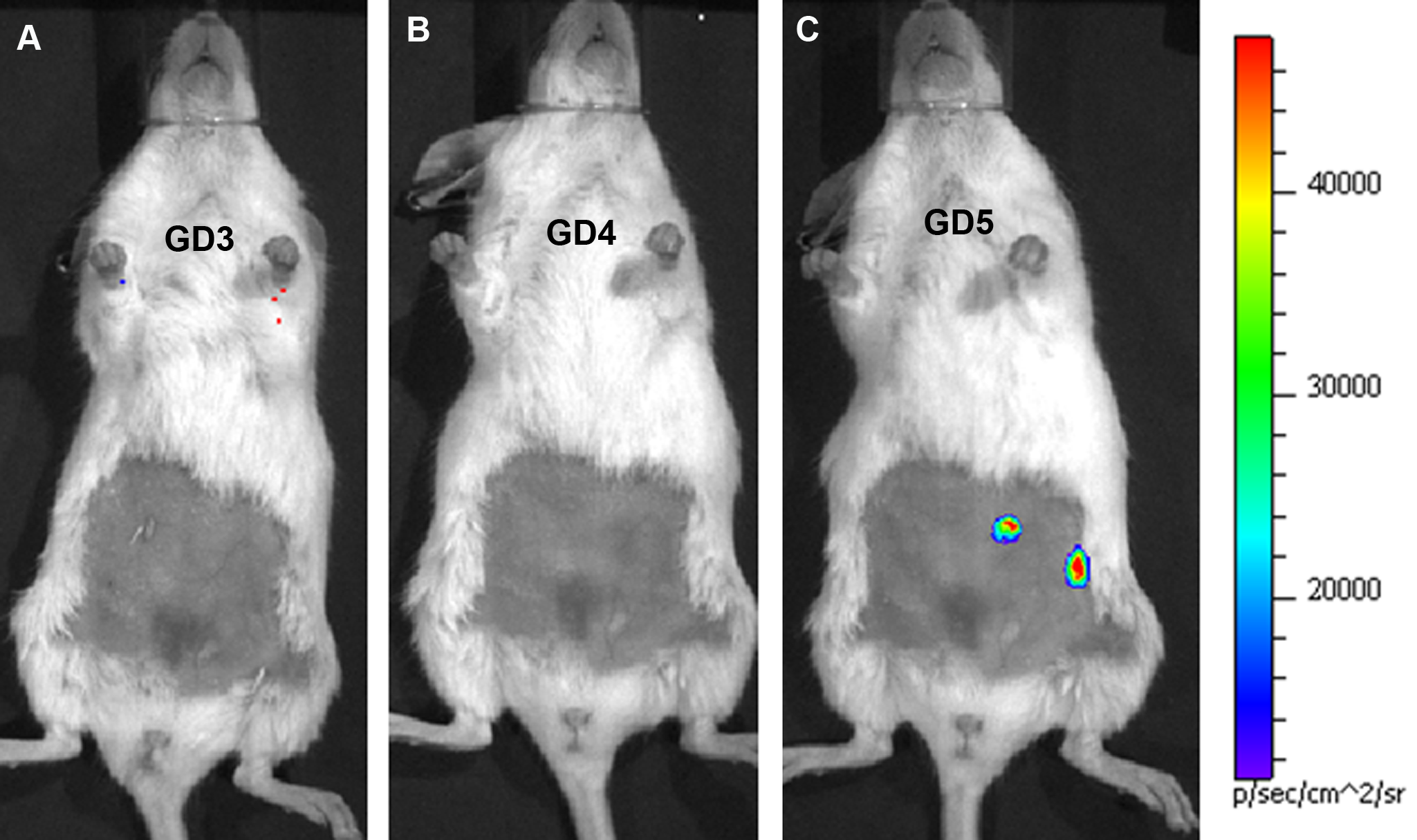

Supplement: Figure S1 — Detection of Fluc expression by live BLI following transfer of LV-Fluc/Tomato-transduced blastocysts with D-luciferin. Blastocysts in M2 medium containing D-luciferin (50 µg/ml) were transferred into GD3 pseudopregnant recipients, and Fluc expression was evaluated after IP injection of D-Luciferin (150 mg/kg body weight) into each animal by live BLI, immediately after blastocyst transfer (GD3 at 2PM, A) and again on GD4 (2PM, B) and GD5 (6PM, C). A–C, superimposed grayscale body surface images and pseudocolor luminescence images. Photons emitted from implanting blastocysts could be detected only on GD5 (C) - there was no detectable signal on GD3 (A) and GD4 (B). (TIF) [file pone.0016348.s001.tif]

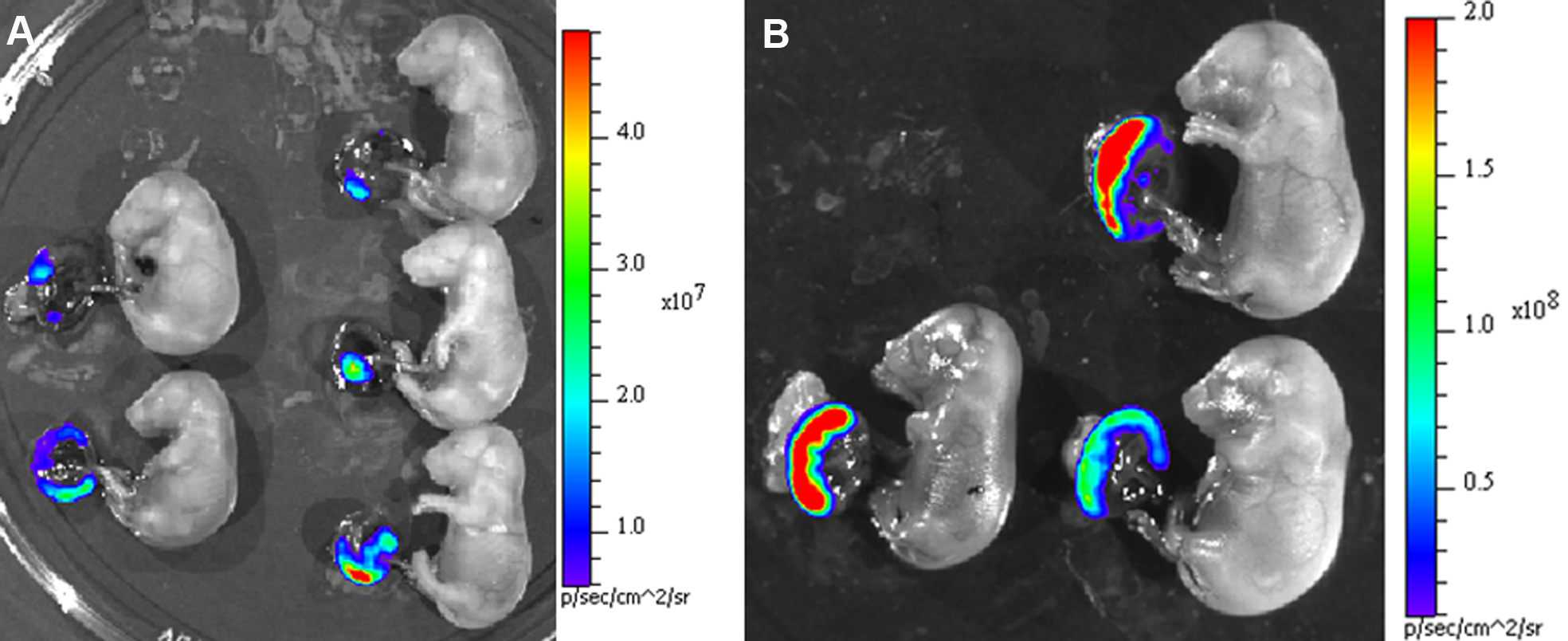

Supplement: Figure S2 — Variability in Fluc expression among placentas of the same litter after transfer of blastocysts with different BLI values. LV-Fluc/Tomato-transduced blastocysts with BLI values in different ranges (1.0E+4 to 4.0E+4, above 3.0E+4, and 2.0E+4 to 6.0E+4 p/s/cm2/sr) were transferred to different groups of GD3 pseudopregnant recipients. A and B, placentas from recipients transferred with blastocysts of BLI values of 1.0E+4 to 4.0E+4 p/s/cm2/sr (A) and above 3.0E+4 p/s/cm2/sr (B). Placentas of transferred blastocysts having BLI values of 2.0E+4 to 6.0E+4 p/s/cm2/sr are presented in Figure 2F. Note that there is wide variability in Fluc expression among placentas in A (1.0E+4 to 4.0E+4 p/s/cm2/sr) and B (above 3.0E+4 p/s/cm2/sr), but not in Figure 2F (2.0E+4 to 6.0E+4 p/s/cm2/sr). (TIF) [file pone.0016348.s002.tif]
